# RNA sequencing reveals a depletion of collagen targeting microRNAs in Dupuytren’s disease

**DOI:** 10.1186/s12920-015-0135-8

**Published:** 2015-10-07

**Authors:** Scott M. Riester, Diren Arsoy, Emily T. Camilleri, Amel Dudakovic, Christopher R. Paradise, Jared M. Evans, Jorge Torres-Mora, Marco Rizzo, Peter Kloen, Marianna Kruithof-de Julio, Andre J. van Wijnen, Sanjeev Kakar

**Affiliations:** Department of Orthopedic Surgery, Mayo Clinic, 200 First Street SW, Rochester, MN 55905 USA; Department of Anatomic Pathology, Mayo Clinic, Rochester, MN USA; Department of Biomedical Statistics and Informatics, Mayo Clinic Rochester, Rochester, MN USA; Department of Orthopedic Surgery, Academic Medical Center, Amsterdam, The Netherlands; Department of Urology, Leiden University Medical Center, Leiden, The Netherlands

**Keywords:** Dupuytren’s disease, microRNA, fibrosis, RNA sequencing, hand

## Abstract

**Background:**

Dupuytren’s disease is an inherited disorder in which patients develop fibrotic contractures of the hand. Current treatment strategies include surgical excision or enzymatic digestion of fibrotic tissue. MicroRNAs, which are key posttranscriptional regulators of genes expression, have been shown to play an important regulatory role in disorders of fibrosis. Therefore in this investigation, we apply high throughput next generation RNA sequencing strategies to characterize microRNA expression in diseased and healthy palmar fascia to elucidate molecular mechanisms responsible for pathogenic fibrosis.

**Methods:**

We applied high throughput RNA sequencing techniques to quantify the expression of all known human microRNAs in Dupuytren’s and control palmar fascia. MicroRNAs that were differentially expressed between diseased and healthy tissue samples were used for computational target prediction using the bioinformatics tool ComiR. Molecular pathways that were predicted to be differentially expressed based on computational analysis were validated by performing RT-qPCR on RNA extracted from diseased and non-diseased palmar fascia biopsies.

**Results:**

A comparison of microRNAs expressed in Dupuytren’s fascia and control fascia identified 74 microRNAs with a 2-fold enrichment in Dupuytren’s tissue, and 32 microRNAs with enrichment in control fascia. Computational target prediction for differentially expressed microRNAs indicated preferential targeting of collagens and extracellular matrix related proteins in control palmar fascia. RT-qPCR confirmed the decreased expression of microRNA targeted collagens in control palmar fascia tissues.

**Discussion:**

Control palmar fascia show decreased expression of mRNAs encoding collagens that are preferentially targeted by microRNAs enriched in non-diseased fascia. Thus alterations in microRNA regulatory networks may play an important role in driving the pathogenic fibrosis seen in Dupuytren’s disease via direct regulatory effects on extracellular matrix protein synthesis.

**Conclusion:**

Dupuytren’s fascia and healthy palmar fascia can be distinguished by unique microRNA profiles, which are predicted to preferentially target collagens and other extracellular matrix proteins.

**Electronic supplementary material:**

The online version of this article (doi:10.1186/s12920-015-0135-8) contains supplementary material, which is available to authorized users.

## Background

Dupuytren’s disease is a clinically challenging disorder characterized by the formation of fibrotic bands that cause disabling contractures of the hand. If the disease is not treated, fibrosis can lead to significant functional limitations that may even necessitate amputation of the affected fingers. Current treatment strategies attempt to break up constrictive bands of fibrous tissue after collagen deposition either surgically or enzymatically with collagenase. These treatments are costly and carry a significant complication risk and are associated with a high rate of disease recurrence [1–5].

Dupuytren’s disease has a strong genetic basis and most commonly affects individuals of northern European descent [6]. Large scale genome wide association studies have helped improve our understanding of Dupuytren’s disease, however the specific genetic abnormalities that drive disease pathogenesis have remained elusive. A variety of molecular pathways have been implicated in disease pathogenesis including alterations in Wnt signaling and mitochondrial genes [7–9].

MicroRNAs, which are small non-coding RNA molecules (20–24 nucleotides in length) that act as post transcriptional regulators of gene expression by inhibiting the translation of target mRNAs, have been shown to regulate the expression of extracellular matrix proteins in the setting of fibrosis [10–13]. Given that Dupuytren’s disease is characterized by excess collagen deposition and fibrosis, we examined the role of microRNAs as pro-fibrotic drivers of the disease process. In this investigation we applied high throughput molecular sequencing techniques to characterize all known microRNAs expressed in diseased Dupuytren’s fascia, and compared expression profiles to non-diseased palmar fascia. We also utilized differentially expressed microRNAs to identify novel pathways as well as validate mechanisms previously implicated with Dupuytren’s disease.

## Methods

### Tissue collection

Dupuytren’s tissue biopsies were collected for research use from patient’s undergoing open palmar fasciectomy for the treatment of Dupuytren’s contracture. Surgical cases clinically represented end stage disease in the consolidation phase. All Dupuytren’s tissue specimens were evaluated under frozen section by trained musculoskeletal pathologists to confirm the diagnosis and to ensure representative areas of diseased tissue were selected. Samples were then snap frozen in liquid nitrogen and stored at −80 °C until use for RNA extraction. Adjacent fascia specimens were obtained from palmar fascia adjacent to diseased Dupuytren’s fascia that was deemed to be normal clinically based upon intraoperative inspection under loupe magnification. To avoid unnecessary risk to patients, adjacent fascia was only collected for research use when sufficient quantities of adjacent tissue were removed during the normal course of surgery, additional surgical procedures were not performed to acquire adjacent fascia. Control palmar fascia biopsies were obtained from healthy patients without a history of Dupuytren’s disease undergoing open carpal tunnel release. Specifically, a small section (~1 cm x 1 cm) of palmar fascia located just superficial to the transverse carpal ligament was collected. The specimen was snap frozen in liquid nitrogen, followed by storage at −80 °C until use for molecular analysis. In total, 25 tissue samples were collected and used for high resolution molecular analysis. There were 15 diseased Dupuytren’s fascia biopsies, seven external controls biopsies, and three adjacent fascia specimens. Informed consent was obtained under institutional review board approved protocols for all specimens used in this investigation (Genetic analysis of disorders of fibrosis IRB # 12–000208).

### RNA extraction

Tissue biopsies were frozen using liquid nitrogen and ground into a powder using a mortar and pestle. Crushed samples were placed into Qiazol reagent and homogenized using the TissueLyser LT (Qiagen, Hilden, Germany). MicroRNAs were extracted from research biopsies using the miRNeasy minikit (Qiagen, Hilden, Germany). Total RNA was quantified using the NanoDrop 2000 spectrophotometer (Thermo Fischer Scientific, Wilmington, Delaware).

### RNA Sequencing (RNA-seq) and bioinformatics analysis

MicroRNAs were sequenced using the NEBNext Small RNA library prep kit on an Illumina HiSeq 2000. The short reads were trimmed of adapters with Cutadapt [14]. Trimmed microRNA sequences greater than 17 nucleotides in length were then aligned to the reference genome and miRBase reference sequences using Bowtie [15]. Known microRNA expression and novel microRNA prediction and quantification were performed with miRDeep2 [16], using the CAP-miRSeq analysis pipeline [17]. Unsupervised hierarchical clustering was performed using the Pearson correlation method. ComiR, a computational tool for combinatorial microRNA target prediction was used to identify molecular pathways regulated by microRNAs that were differentially expressed between diseased and non-diseased palmar fascia [18, 19]. The Database for Annotation and Visualization and Integrated Discovery v6.7 (DAVID 6.7) [20, 21] was used to characterize functional gene clusters regulated by microRNA target genes.

### RT-qPCR validation

The activity of pro-fibrotic pathways, including the Wnt and TGFβ signaling pathways, were assessed by measuring the expression of regulatory mRNAs using real-time quantitative polymerase chain reaction (RT-qPCR). Total RNA from 11 Dupuytren’s specimens and seven control biopsies, which were previously used for small RNA-seq, were used for cDNA synthesis. Reverse transcription and RT-qPCR reactions were performed as previously described by Dudakovic et al. [22]. Transcript levels were normalized to AKT1, because this gene is most consistently expressed across samples within the mesenchymal lineage compared to other conventional housekeeping genes including GAPDH, HPRT and ACTB based on data we obtained for >400 different mesenchymal cell types and musculoskeletal tissues (SMR & AJvW, unpublished data). Gene expression levels were quantified using the 2^-∆∆Ct^ method. Differences in gene expression between diseased and control samples were evaluated using a two-tailed student’s *t*-test. Error bars are shown as the mean ± one standard deviation statistical significance was set at *p* < 0.05 and is indicated by (*). Primer sequences are given in (Additional file 1: Table S1).

## Results

### RNA-seq evaluation

**Fig. 1:**
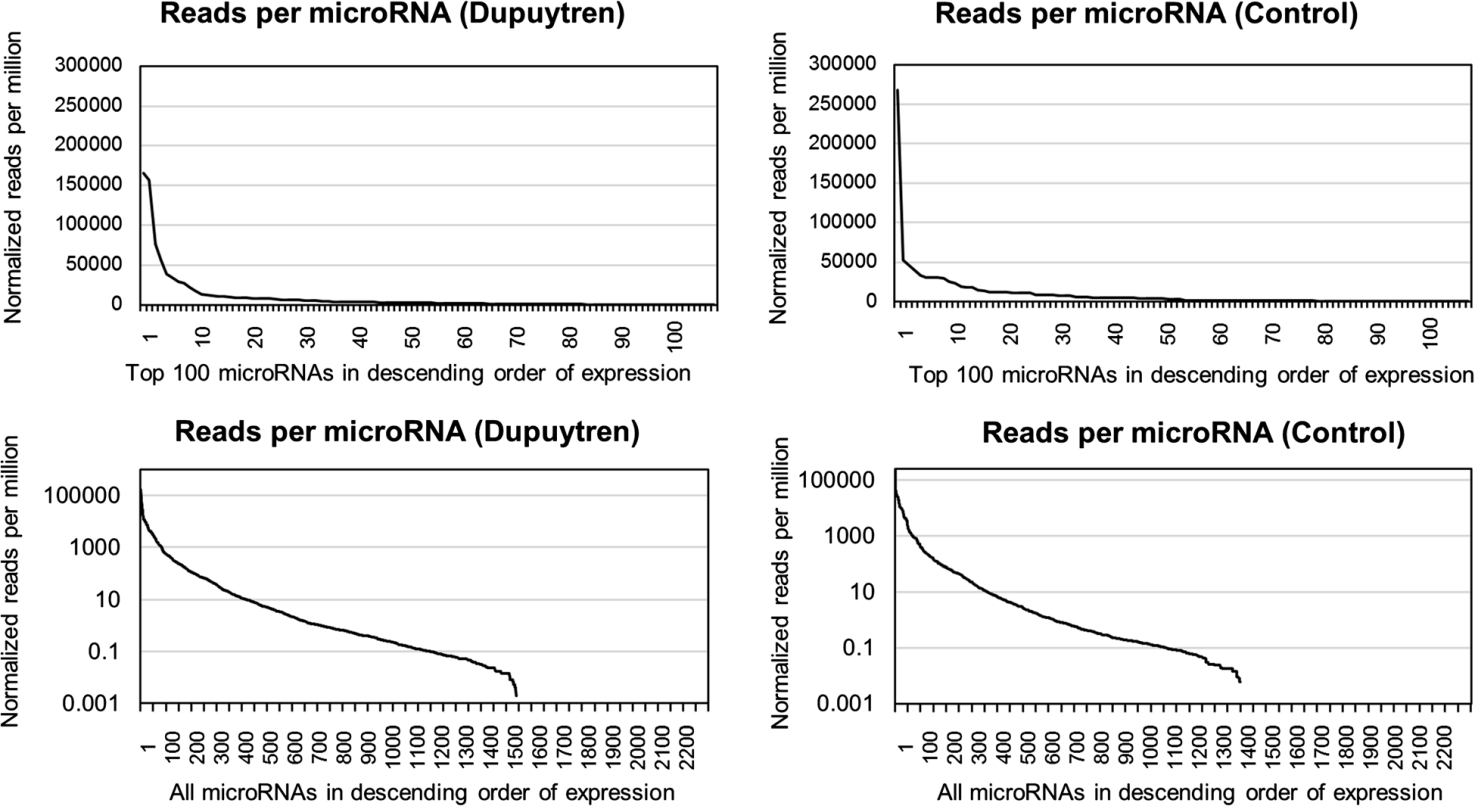
MicroRNA sequencing reads are shown plotted on a linear (top) and log scale (bottom) for both Dupuytren’s and control palmar fascia tissue samples. These plots show that the top 100 most highly expressed microRNAs account for greater than 90 % of the total sequence reads. Thus, a small subset of microRNAs are expressed at a much higher levels than all the remaining human microRNAs, and may have a greater functional impact on cell activity

In this investigation we applied high throughput next generation RNA-seq to quantify the expression of all known human microRNAs (*N* = 2252) to understand their roles in the pathogenesis of Dupuytren’s disease. Initial sequencing results showed that more than 99 % of the sequencing reads averaged across all Dupuytren’s and control samples were represented by the top 100 most abundantly expressed microRNAs (Fig. 1). This distribution is consistent across both the diseased and control tissues, and shows that a relatively small subset of all known human microRNAs account for the majority of microRNAs expressed in palmar fascia biopsies. These abundant microRNAs may therefore play a more important functional role in regulating cellular processes connected to Dupuytren’s disease pathogenesis than microRNAs with low levels of expression.

**Fig. 2:**
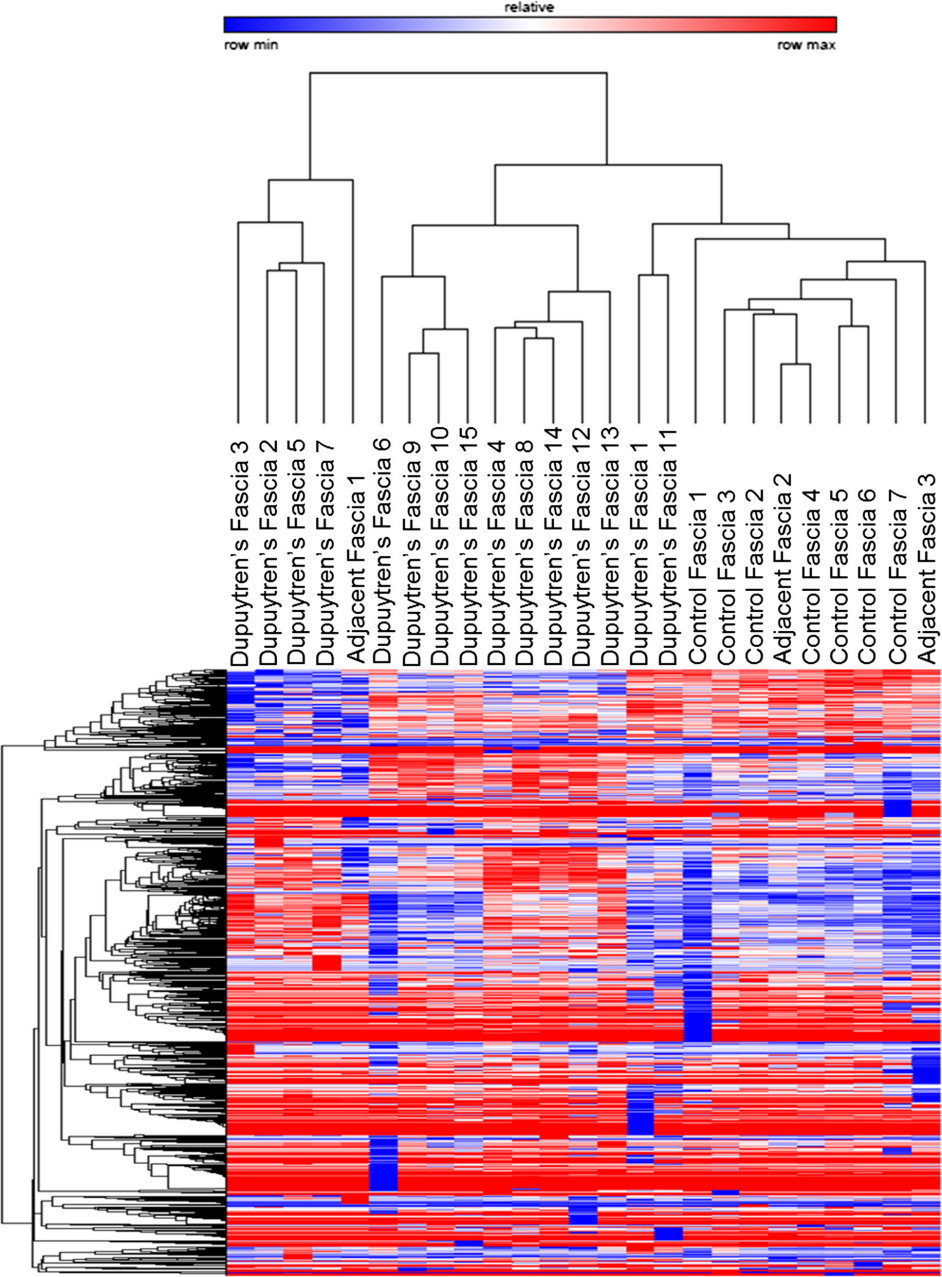
Unsupervised hierarchical clustering of comprehensive microRNA sequencing reads using the Pearson correlation method results in independent clustering of Dupuytren’s and control palmar fascia. Only a single adjacent fascia specimen clustered with the Dupuytren’s fascia indicating that visual inspection alone may not be reliable in distinguishing between diseased and non-diseased fascia. These data show that microRNA expression patterns may be able to differentiate between diseased and non-diseased fascia and may play key roles in driving pathogenic fibrosis

Unsupervised hierarchical clustering was performed using the Pearson correlation method to provide an unbiased assessment of the ability of microRNAs to differentiate between diseased and non-diseased palmar fascia biopsies (Fig. 2). The clustering dendrogram showed independent grouping of the control and diseased palmar fascia biopsies. There was only a single adjacent fascia biopsy that clustered with the Dupuytren’s specimens suggesting that this specimen may have in fact been diseased tissue that appeared grossly normal intra-operatively.

**Fig. 3:**
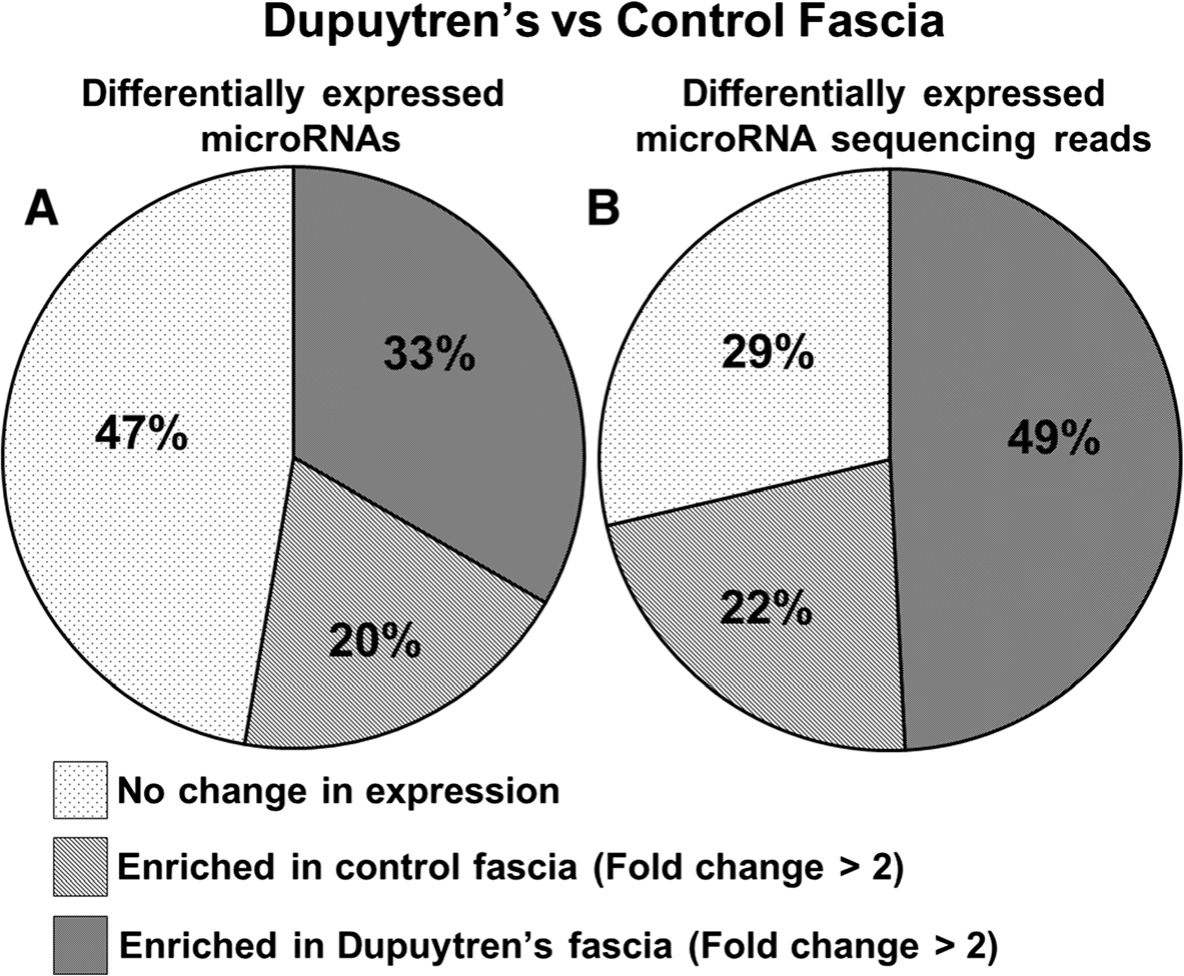
A comparison of the absolute number of microRNAs and corresponding sequencing reads enriched in Dupuytren’s versus control fascia for abundantly expressed microRNAs (microRNAs expressed at more than 100 normalized reads per million). These data show that Dupuytren’s tissue has greater enrichment in different types of microRNAs (**a**) as well as the absolute number of microRNAs based on normalized read count (**b**)

**Fig. 4:**
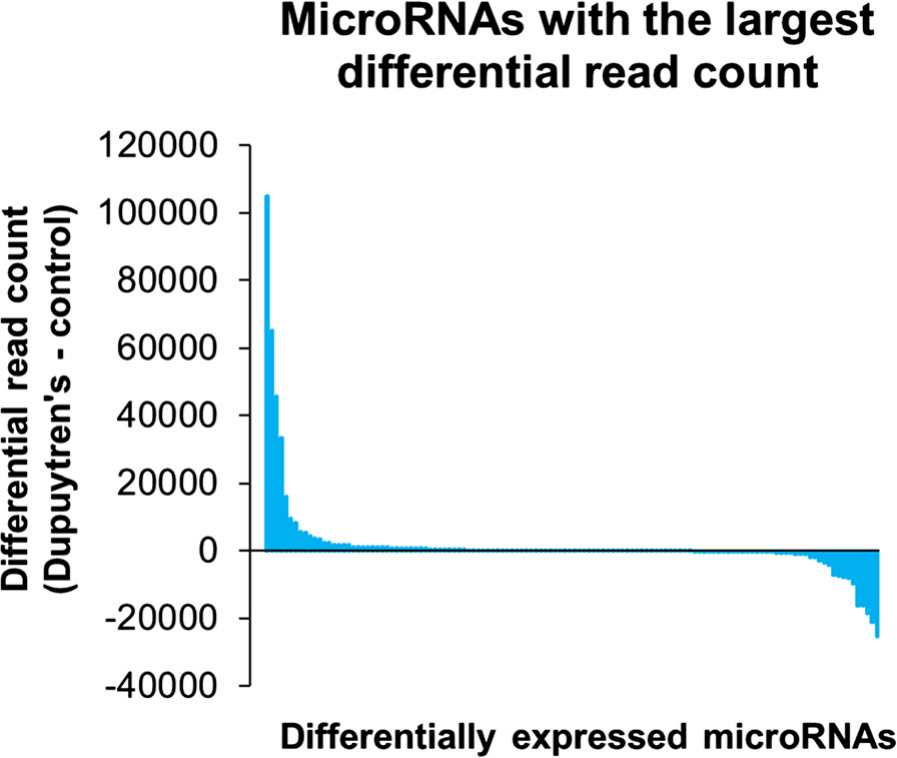
To identify the microRNAs with the greatest functional impact on fibrosis, we examined which microRNAs show the greatest difference in sequencing reads between diseased Dupuytren’s fascia and control fascia regardless of fold change. This analysis, comparing all statistically significant differentially expressed microRNAs with an average expression greater than 100 normalized reads, shows that a small subset of differentially expressed microRNAs account for the majority of sequencing reads. These microRNAs, which show the greatest change in abundance, may potentially make the greatest functional contribution to the phenotypic differences observed between Dupuytren’s fascia and non-diseased fascia

To identify specific microRNAs with a functional role in the pathogenesis of Dupuytren’s disease we made a fold change comparison of microRNA profiles in Dupuytren’s biopsies against control samples (excluding adjacent fascia). In the comparison of Dupuytren’s fascia and control fascia we identified 74 microRNAs with a statistically significant 2-fold enrichment in Dupuytren’s tissue, and 32 microRNAs with a statistically significant enrichment in control fascia (Additional file 2: Table S2). The diseased Dupuytren’s fascia show enrichment (fold change > 2) in a greater variety of different microRNAs compared with control fascia (Fig. 3a), and an even larger increase in the overall number of microRNA sequencing reads (Fig. 3b). To determine if the differentially expressed sequencing reads were being generated by a large number of different microRNAs, or a focused group of microRNAs we examined the distribution of differentially expressed microRNA sequencing reads. The majority of the differentially expressed microRNA reads were found to be concentrated amongst a relatively small group of differentially expressed microRNAs (Fig. 4). Notably the microRNAs upregulated in the control fascia accounting for the greatest differential in read count are heavily enriched in previously validated anti-fibrotic extracellular matrix targeting microRNAs (Table 1), including let-7 [23–25], miR-29a-3p [26], miR-26b-5p, miR-30d-5p [27, 28], miR-27a-3p, miR-27b-3p [29, 30], miR-10a-5p [31], miR-26a-5p [32–35], miR-101-3p [36–39], and miR-10b-5p [40], as well as anti-proliferative microRNAs including, miR-126-3p [41–47], miR-99a-5p [48–54], miR-125a-5p [55–59], and miR-139-5p [60–62]. MicroRNAs enriched in Dupuytren’s fascia target appear to target a diverse array of functional pathways. miR-21-5p is an established oncomiR that promotes cellular proliferation in cancer [63–65]. miR-210 is a known hypoxia inducible, fibrosis promoting microRNA that may be associated with decreased tissue perfusion in Dupuytren’s cords [66, 67]. These findings suggest that a relatively small group of microRNAs with large differences in expression act to selectively target molecular pathways regulating tissue fibrosis.

**Table 1:** MicroRNAs differentially expressed between Dupuytren’s and control palmar fascia

MicroRNAs enriched in Dupuytren’s fascia		MicroRNAs enriched in external control fascia	
microRNA	Differential read count	microRNA	Differential read count
	Dupuytren’s vs external control fascia		External control vs Dupuytren’s fascia
hsa-miR-21-5p	104875.60	hsa-miR-10b-5p	25385.58
hsa-miR-320a	65390.83	hsa-miR-26a-5p	21275.22
hsa-miR-22-3p	45760.50	hsa-miR-126-3p	18657.06
hsa-miR-378a-3p	33330.36	hsa-let-7 g-5p	16538.33
hsa-let-7b-5p	16107.56	hsa-miR-99a-5p	16257.13
hsa-miR-134	9518.18	hsa-miR-101-3p	9984.21
hsa-miR-127-3p	8325.92	hsa-let-7f-5p	8123.18
hsa-miR-23a-3p	5829.286	hsa-let-7a-5p	7884.75
hsa-miR-370	5474.795	hsa-miR-27a-3p	7553.27
hsa-miR-378c	4531.18	hsa-miR-27b-3p	7262.95
hsa-miR-152	3644.90	hsa-miR-30d-5p	4436.03
hsa-miR-145-5p	3469.33	hsa-miR-29a-3p	3611.05
hsa-miR-181a-5p	2433.00	hsa-miR-125a-5p	3042.29
hsa-miR-381-3p	2361.47	hsa-miR-10a-5p	2033.13
hsa-miR-452-5p	1804.42	hsa-miR-26b-5p	1971.30
hsa-miR-409-3p	1804.10	hsa-let-7d-5p	1153.14
hsa-miR-127-5p	1708.96	hsa-miR-139-5p	1096.97
hsa-miR-146b-5p	1662.59		
hsa-miR-23b-3p	1281.21		
hsa-miR-193b-3p	1275.18		
hsa-miR-320b	1148.60		
hsa-miR-146a-5p	1135.37		
hsa-miR-210	1055.20		
hsa-miR-708-3p	1027.45		
hsa-miR-769-5p	1004.10		
hsa-miR-378d	1000.99		

**Fig. 5:**
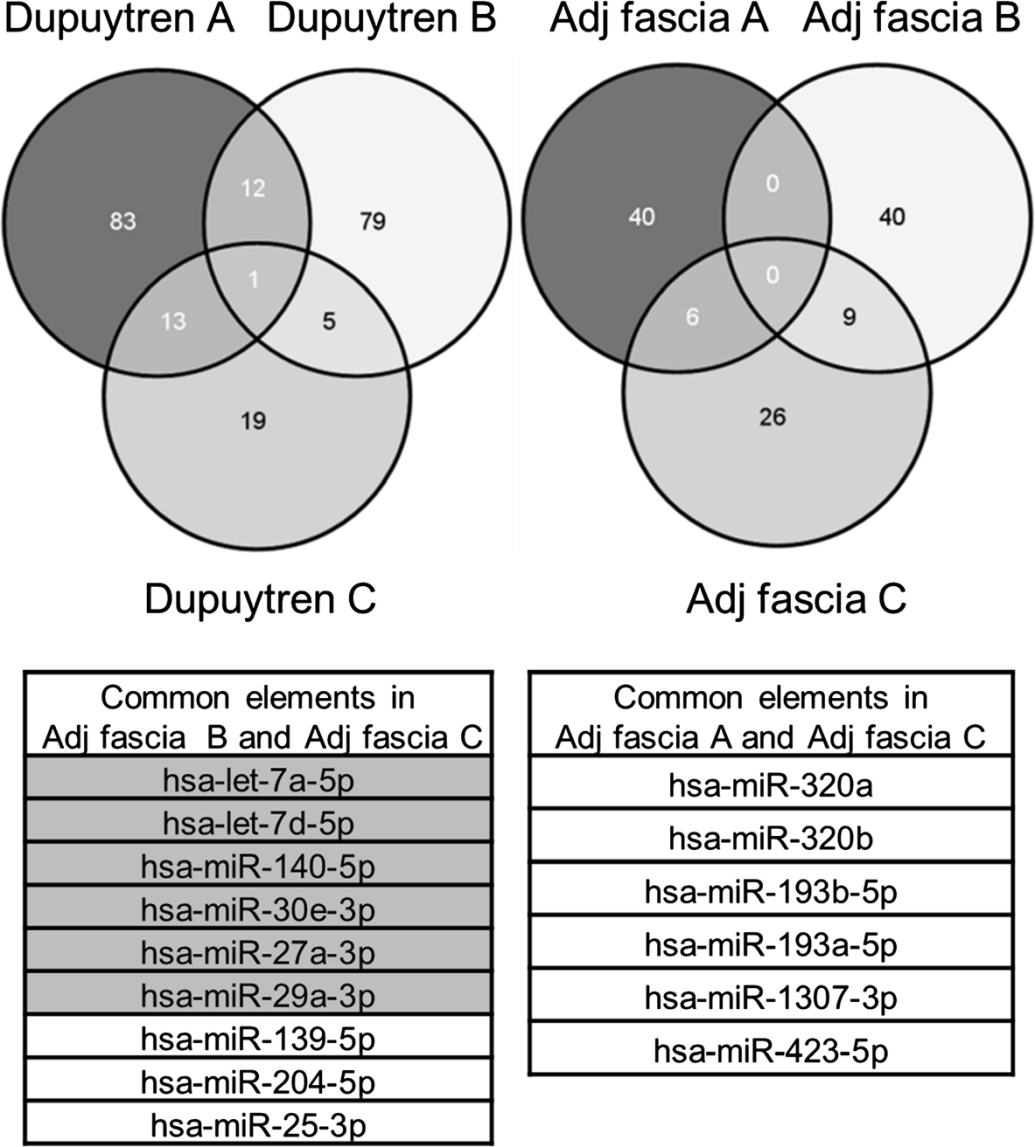
A venn diagram showing a comparison of microRNAs upregulated (fold change > 2) in patient matched Dupuytren’s fascia and adjacent fascia biopsies. It is notable that there are no microRNAs commonly upregulated in any of the patient matched biopsies in either the Dupuytren’s or the adjacent fascia, highlighting the heterogeneity (Dupuytren’s vs control phenotype) of the adjacent fascia biopsies. During unsupervised hierarchical clustering analysis (Fig. 2) specimen “**a**” preferentially clustered with Dupuytren’s fascia, thus intraoperative examination of surgical tissue may not be able to reliably differentiate between diseased and non-diseased fascia at the molecular level. It is notable that adjacent fascia specimens “**b**” and “**c**”, which cluster with control fascia, both show enrichment in known collagen targeting microRNAs (highlighted in grey), which are depleted in specimen “A”

To determine if adjacent fascia exhibits a disease-like molecular phenotype, we compared the adjacent fascia biopsies with patient matched diseased Dupuytren’s fascia. A direct comparison of the three matched adjacent fascia and Dupuytren’s fascia biopsies revealed miR-181b-5p as the only microRNA with a statistically significant enrichment greater than 1.4 fold in the Dupuytren’s fascia. The lack of statistically significant microRNAs is attributable largely to the variability in the adjacent fascia biopsy specimens (Dupuytren vs control phenotype); therefore we compared upregulated microRNAs in each paired sample using a Venn diagram method (Fig. 5). This analysis showed an enrichment of anti-fibrotic extracellular matrix targeting microRNAs in two of the adjacent fascia specimens relative to their matched Dupuytren’s biopsy [23–29, 68, 69]. However the single adjacent fascia specimen that clustered with the diseased Dupuytren’s fascia during unsupervised hierarchical clustering (specimen “A”) did not show enrichment in collagen/extracellular matrix targeting microRNAs. These findings support its preferential clustering with the Dupuytren’s fascia and indicate that its molecular phenotype more closely resembles that of diseased fascia rather than control fascia despite its grossly normal appearance. A comparison of adjacent and control fascial biopsies also did not reveal any microRNAs with a statistically significant enrichment in the adjacent fascia, again highlighting the variability of the adjacent fascia biopsies. There were however seven microRNAs (miR-335-3p, miR-128, miR-224-5p, miR-28-5p, miR-191-5p, miR-423-3p, and miR-181a-5p) with a statistically significant upregulation in the controls. Two of these seven microRNAs, miR-335-3p, and miR-181a-5p are also validated extracellular matrix targeting microRNAs [30, 31, 70–72]. Their enrichment in external control specimens supports their previously described role as anti-fibrotic microRNAs, and is a reflection of a partial disease phenotype in the adjacent fascia specimens. These findings highlight the potential utility of microRNAs as biomarkers to confirm diagnosis and define optimal surgical margins to ensure all diseased fascia is removed at the time of surgery. It also implicates microRNAs as potential therapeutic targets/agents to prevent as well as treat active fibrotic disease.

### Computational microRNA target prediction

MicroRNAs have the ability to inhibit a large number of target genes by binding to sequence specific 3’UTR regions of target mRNAs, inhibiting their translation and promoting their degradation [73]. To confirm preferential targeting of pathways linked to extracellular matrix synthesis and cellular proliferation, and to identify novel pathways that are regulated by microRNAs in Dupuytren’s disease, we performed computational target prediction using the combinatorial miRNA target prediction tool ComiR [18, 19] (Additional file 3: Table S3). This program uses computational targets generated using miRanda, PITA, TargetScan, and mirSVR, and determines gene targets for a set of microRNAs taking into account the relative expression of each microRNA in a set of samples. We compared microRNA gene targets between Dupuytren’s fascia, and control fascia only, since adjacent fascia may represent an intermediate state between Dupuytren’s tissue and unaffected external control tissue. Abundant, differentially expressed microRNAs with an average expression level of at least 100 normalized reads per million in either the Dupuytren’s or control specimens were evaluated.

**Fig. 6:**
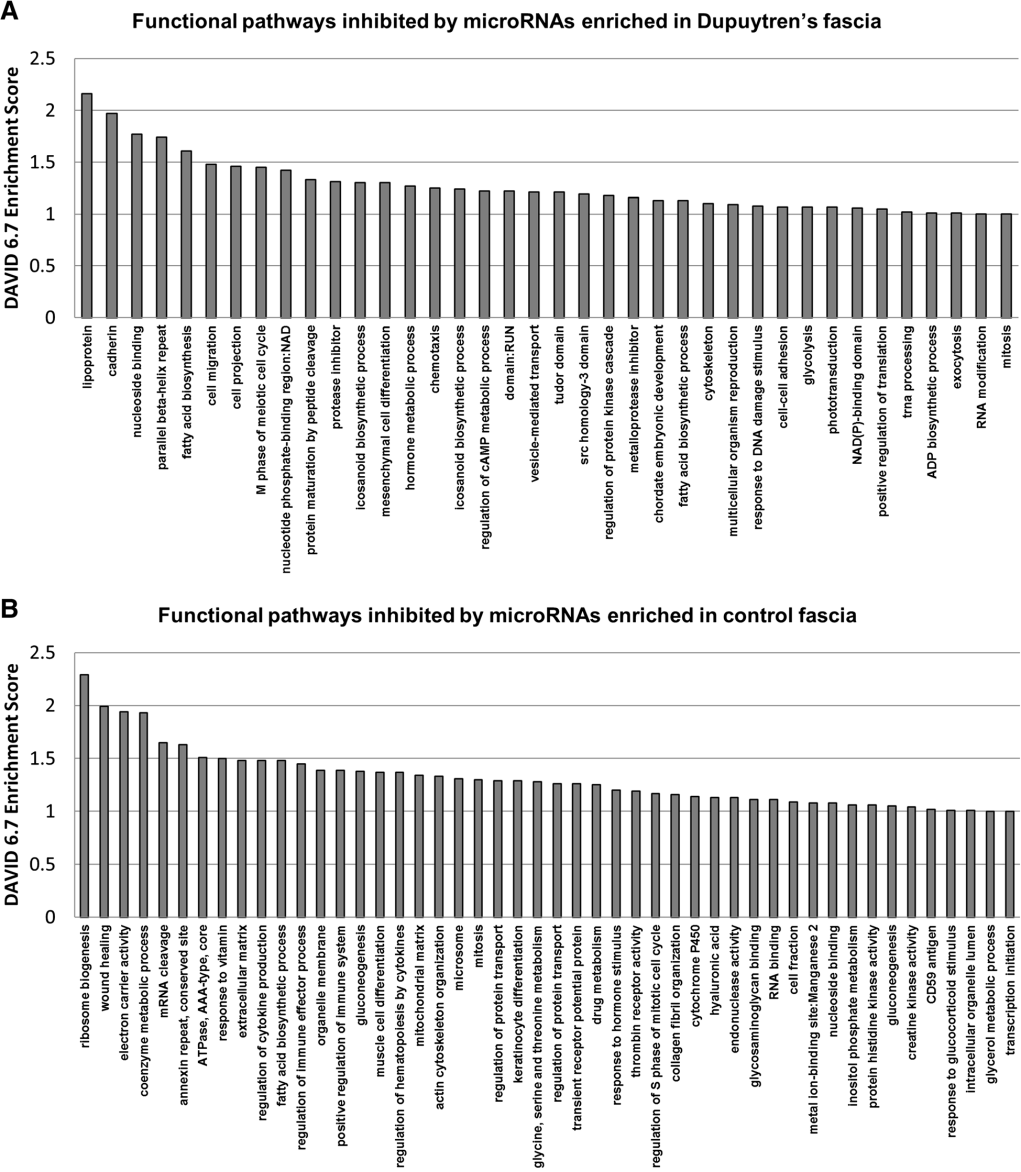
**a** Functional annotation clustering using DAVID 6.7 was performed for microRNA genes targets enriched in Dupuytren’s fascia using ComiR target prediction. Functional gene clustering shows preferential targeting and inhibition of pathways linked to lipid synthesis and metalloprotease inhibitors in diseased Dupuytren’s fascia. **b** Functional annotation clustering using DAVID 6.7 was performed for microRNA genes targets enriched in control palmar fascia using ComiR target prediction. Functional gene clustering shows preferential targeting and inhibition of pathways linked to collagen and extracellular matrix synthesis, inflammation and immune response, as well as cell division and mitosis in non-diseased control palmar fascia

Analysis with ComiR identified 6685 genes that were computationally predicted to be inhibited by microRNAs enriched in control fascia (*p* < 0.05). In the Dupuytren’s fascia there were 4969 genes computationally predicted to be targeted by enriched microRNAs. Functional annotation clustering using David 6.7 for the top 3000 most heavily targeted genes in control and diseased fascia identified candidate molecular pathways regulated by microRNAs (Fig. 6). ComiR analysis confirmed preferential targeting of extracellular matrix synthesis and cellular proliferation in the control palmar fascia. Notable microRNA inhibited pathways in control fascia included wound healing (enrichment score 1.99), extracellular matrix synthesis (enrichment score 1.48), cytokine production (enrichment score 1.48), mitochondrion (enrichment score 1.39), muscle cell differentiation (enrichment score 1.37), cell cycle (enrichment score 1.3), epithelial cell differentiation and keratinization (enrichment score 1.29), and collagen and laminin production (enrichment score 1.16). In diseased Dupuytren’s fascia, microRNAs are most notably predicted to inhibit pathways linked to lipid-binding/lipoproteins (enrichment score 2.16), cadherins (enrichment score 1.97), fatty acid biosynthesis (enrichment score 1.61), and metalloenzyme inhibitors (enrichment score 1.16).

### RT-qPCR validation of microRNA gene targets

**Fig. 7:**
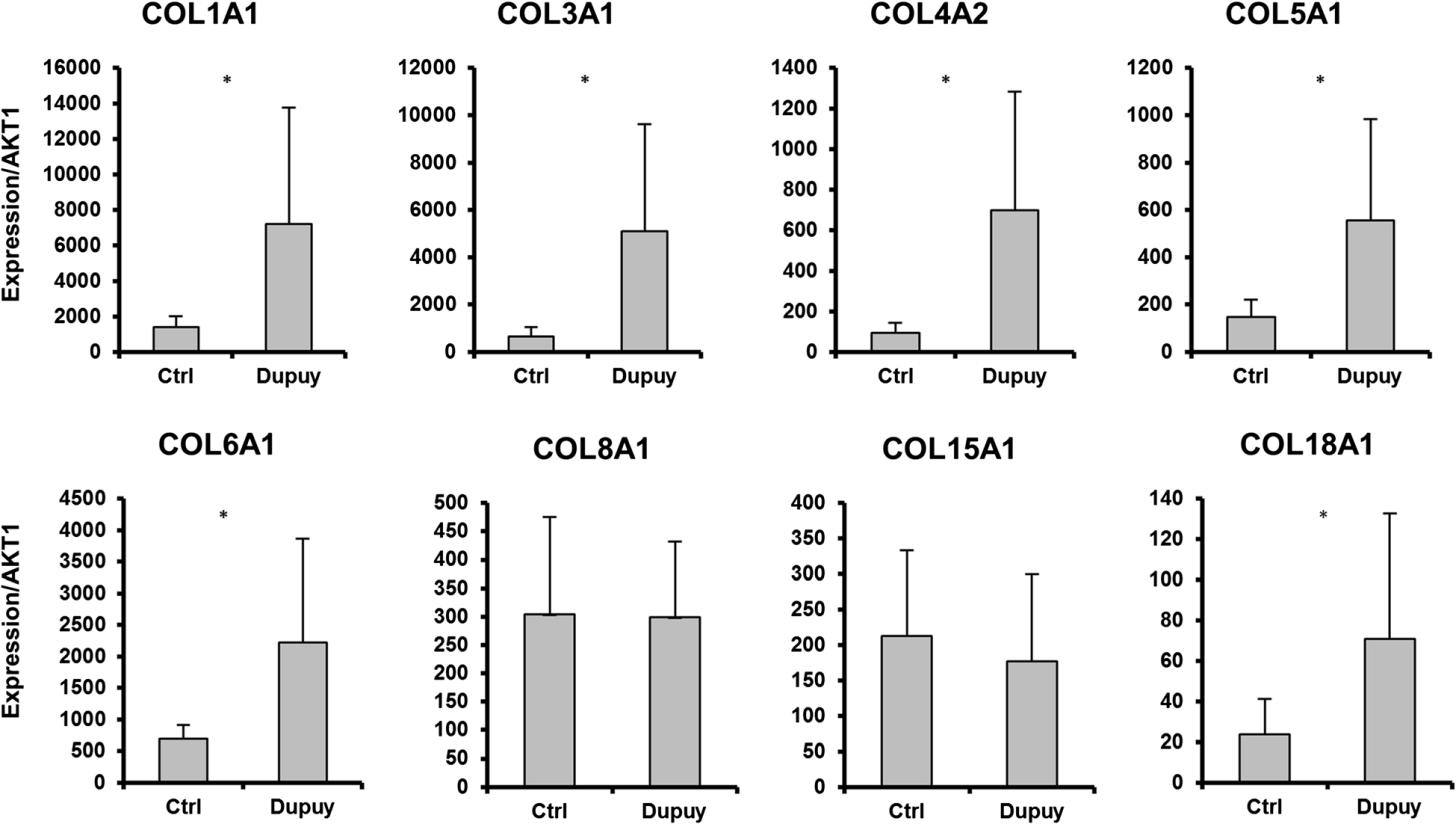
RT-qPCR evaluation of ten selected collagens in Dupuytren’s and control palmar fascia are shown. COL8A1 and COL15A1 are the only collagens that do not show a statistically significant enrichment in Dupuytren’s fascia. These findings are in accordance with ComiR predictions, which show that these two exhibit the lowest degree of inhibitory microRNA targeting in control fascia

Since extracellular matrix synthesis is predicted to be inhibited by microRNAs enriched in control palmar fascia, we examined the expression of collagens, the primary constituents of extracellular matrix in palmar fascia. We performed RT-qPCR to evaluate the expression of selected collagens linked to fibrosis in diseased and non-diseased tissues (Fig. 7). We then examined which of these collagens were preferentially targeted for microRNA inhibition in control palmar fascia based on ComiR analysis (Table 2). RT-qPCR of fibrosis related collagens shows that collagens predicted to be targeted by microRNA inhibition in controls are enriched in Dupuytren’s fascia, while collagens with less microRNA targeting (e.g. COL15A1 and COL18A1) do not show statistically significant differences in expression between diseased and non-diseased tissue. These results show that the expression of collagen targeting microRNAs inversely correlates with the expression of fibrosis related collagens in palmar fascia.

**Table 2:** Collagens targeted by microRNAs enriched in control palmar fascia - ComiR target prediction shows that the majority of collagens are preferentially inhibited by microRNAs enriched in control fascia compared with Dupuytren’s fascia. In our analysis of collagen expression using RT-qPCR, COL8A1 and COL15A1 were the only collagens that did not show a statistically significant increase in Dupuytren’s fascia. Notably these collagens also exhibited the lowest degree of microRNA targeting

GENE ID	ComiR score difference (Control Fascia –Dupuytren’s Fascia)	*p*-value
COL5A3	0.0896	0.0007
COL1A2	0.0804	<0.001
COL4A2	0.0630	<0.001
COL11A1	0.0602	<0.001
COL3A1	0.0543	<0.001
COL5A1	0.0472	0.0015
COL18A1	0.0469	0.0033
COL1A1	0.0431	<0.001
COL13A1	0.0407	0.0488
COL21A1	0.0387	<0.001
COL15A1	0.0386	0.0005
COL5A2	0.0381	0.0006
COL7A1	0.0344	<0.001
COL12A1	0.0330	0.0749
COL16A1	0.0192	0.0028
COL4A4	−0.0031	0.1286
COL27A1	−0.0103	0.3310
COL8A1	−0.0136	0.1502
COL2A1	−0.0186	0.0141
COL4A1	−0.0187	0.3535
COL6A1	−0.0238	0.0715
COL6A3	−0.0245	0.0523
COL8A2	−0.0463	<0.001
COL6A2	−0.1577	<0.001

**Fig. 8:**
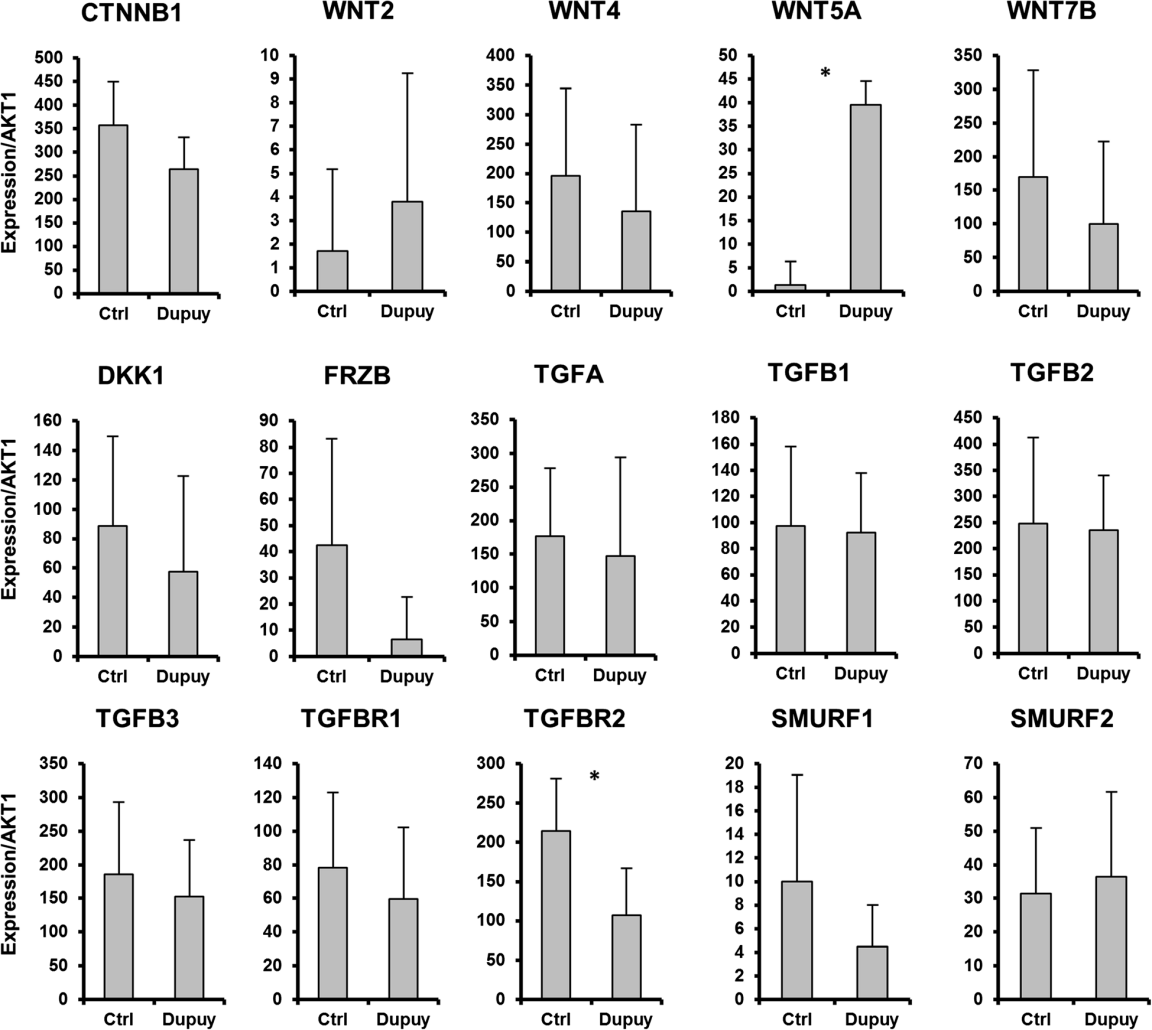
A RT-qPCR screen for fibrosis associated mRNAs linked to Wnt and TGFβ signaling are shown. This analysis reveals a statistically significant upregulation of WNT5A in Dupuytren’s fascia, and a paradoxical upregulation of TGFBR2 in control fascia. WNT5A is predicted to be downregulated by microRNAs enriched in Dupuytren’s disease based on ComiR analysis, thus WNT5A upregulation is unlikely to be mediated solely by microRNA regulatory. In accordance with ComiR target predictions, we do not observe strong evidence for direct microRNA regulation of TGFβ and Wnt signaling pathways in Dupuytren’s disease

Previous studies have implicated alterations in Wnt and TGFβ signaling in the pathogenesis of Dupuytren’s disease [7, 74, 75]. To determine if microRNAs regulate either of these pathways, we evaluated the expression of mRNAs associated with these two signaling pathways using RT-qPCR (Fig. 8). TGFBR2 and WNT5A were the only two genes to show statistically significant differences in expression between the Dupuytren’s and control palmar fascia. TGFBR2 paradoxically showed increased expression in the control fascia, enrichment inconsistent with its known fibrosis promoting affects. WNT5A was the only gene to show a statistically significant enrichment in the Dupuytren’s fascia. However microRNA target prediction indicates that WNT5A is preferentially inhibited by microRNAs enriched in Dupuytren’s fascia, indicating that microRNA regulation is unlikely to be driving the increased expression of WNT5A in diseased palmar fascia. These findings suggest that microRNAs, which promote mRNA degradation, do not play a major role in post-transcriptional regulation of WNT and TGFβ signaling pathways in the terminal stages of Dupuytren’s disease.

## Discussion

Previous studies using microarrays, which give relative abundance of mRNAs and microRNAs, have been used to study differences in microRNA expression between Dupuytren’s and non-diseased palmar fascia [76, 77]. In comparison with microarrays, RNA-seq provides a broader dynamic range for accurate quantification of differentially expressed transcripts [78]. Therefore in this investigation we applied RNA-seq technology to quantify the expression of all known human microRNAs in Dupuytren’s and control palmar fascia biopsies. Our initial assessment of microRNA sequencing data from the Dupuytren’s and control palmar fascia biopsies using unbiased unsupervised hierarchical clustering confirmed previous findings with microarray analysis, showing that diseased and non-diseased specimens cluster based upon their microRNA profiles [76, 77].

In comparison with our study, Mosakhani et al. applied microarrays to evaluate microRNA expression in Dupuytren’s tissue samples [77]. Our studies confirmed enrichment of microRNAs miR-10b, miR-7f, miR-101, miR-26a, miR-26b, miR-29a, and miR-30 in non-diseased palmar fascia samples. A large number of microRNAs identified as being enriched in either the Dupuytren’s or control fascia were not found to be statistically significant in our analysis. Interestingly, miR-21 the most abundant microRNA in all samples from our study, in contrary to their results, was found to be enriched in Dupuytren’s samples rather than control samples. These differences may be attributable to the fact that microarrays can become saturated with abundantly expressed transcripts, making fold change comparisons unreliable. Additional considerations include the use of fewer control samples (*N* = 4), and the fact that each of the control samples were taken from the transverse carpal ligament, which is deep to the true palmar fascia which gives rise to the Dupuytren’s cords. Two of the control samples were also collected from patients with acute hand trauma, which could also significantly alter microRNA profiles. Satish et al. compared transcriptomes of fibroblasts derived from Dupuytren’s fascia, palmar fascia, and the transverse carpal ligament using microarrays. They found that fibroblasts from the Dupuytren’s fascia and palmar fascia were more similar to one another than either one was to the transverse carpal ligament derived fibroblasts [76]. Thus the comparisons used in our study comparing palmar fascia to Dupuytren’s fascia are likely to be highly informative.

The microRNAs identified in this investigation that are enriched in control fascia encompass known, as well uncharacterized, but potentially novel anti-fibrotic microRNAs. Established anti-fibrotic microRNAs identified in our analysis include let-7 [23–25], miR-29a-3p [26], miR-26b-5p, miR-30d-5p [28, 29], miR-27b-3p [30, 31], miR-10a-5p [33], miR-26a-5p [37–40], miR-101-3p [41–44], miR-27a-3p and miR-10b-5p [45]. Additional enriched microRNAs (miR-126-3p [46–52], miR-99a-5p [53–59], miR-125a-5p [60–64], and miR-139-5p [65–67]) have been shown to affect proliferation in cancer, and may regulate the fibroproliferative activity seen in Dupuytren’s disease. Synergistic activation or inhibition of these microRNAs, which will be investigated in future studies, may collectively permit the potent attenuation or activation of fibrosis for therapeutic applications.

Previous studies have looked at single microRNA targets, and have implicated microRNAs as regulatory factors in Wnt and TGFβ signaling. In this study we used a comprehensive approach simultaneously taking into account the microRNA targets for all abundant, differentially expressed microRNAs. This analysis suggests a loss of microRNAs that target extracellular matrix synthesis in Dupuytren’s disease. MicroRNA target prediction also showed strong correlation with mRNA expression as demonstrated by the expression of extracellular matrix forming collagens. In contrast to previous studies, we did not observe strong evidence for microRNA regulation of Wnt or TGFβ signaling pathways. This finding was also exemplified by RT-qPCR expression data that did not show major difference in the expression of gene transcripts implicated in either of these signaling pathways. These findings do not rule out the possibility of post translational mechanisms (e.g. protein phosphorylation) in propagating either of these pathways. It is also important to note that the tissues examined in this investigation are from patients with advanced disease requiring surgical resection. Collagen synthesis and deposition is characteristic of late stage Dupuytren’s disease where the diseased cords are in the process of consolidating. These studies do not exclude the possibility that earlier stages of disease may still be mediated by over activation of Wnt or TGFβ signaling pathways.

Our analytical approach takes into account the fact that large changes in the expression of a small group of microRNAs may have a more dramatic change on cellular phenotype, than small changes in a large number of different microRNAs because of the lack of coordinated mRNA inhibition by differentially expressed microRNAs. This study also supports the concept that abundantly expressed microRNAs are well suited for fine tune regulation of genes translated from plentiful transcripts such as collagens and other extracellular matrix constituents. Since extracellular matrix targeting microRNAs are constitutively present in large quantities, they can act as a buffer to fine tune extracellular matrix synthesis. This is in stark contrast to alternative regulatory elements such as transcription factors that produce an all or nothing response by directly activating or suppressing mRNA transcription. Analysis of the impact of broad spectrum microRNA targeting, rather than an evaluation of their effects on individual target genes may give greater insight into the important role that microRNAs play in regulating cellular processes.

## Conclusions

The main finding of this study is that microRNA profiles show distinct expression patterns that differentiate diseased Dupuytren’s and healthy palmar fascia. The microRNAs enriched in healthy tissue show preferential targeting of collagens and extracellular matrix proteins. This finding is strongly supported by the fact that differential collagen expression as determined by RT-qPCR, is strongly related to the degree of predicted microRNA targeting.

The microRNAs characterized in this investigation have the potential to serve as disease biomarkers that can help guide surgical management by determining optimal surgical margins during open fasciectomy. Novel RNA-therapeutics that are currently in development, also have the potential to target disease specific microRNAs and prophylactically prevent disabling fibrosis minimizing the need for invasive surgical treatments. Fibrosis related microRNAs may also play important regulatory roles in other disorders of fibrosis including scleroderma, idiopathic pulmonary fibrosis, as well as scarring and wound healing.

## Additional files

Additional file 1: Table S1. Primer sequences used for RT-qPCR evaluation of gene expression in this investigation. (XLSX 10 kb) Additional file 2: Table S2. MicroRNAs showing a 2-fold, or greater statistically significant differences in expression between Dupuytren’s and control palmar fascia. (XLSX 13 kb) Additional file 3: Table S3. ComiR scores for microRNAs showing the greatest difference in expression based on total read count. (XLSX 4350 kb)
