# Erratum to: RNA sequencing reveals a depletion of collagen targeting microRNAs in Dupuytren’s disease

**DOI:** 10.1186/s12920-016-0199-0

**Published:** 2016-07-13

**Authors:** Scott M. Riester, Diren Arsoy, Emily T. Camilleri, Amel Dudakovic, Christopher R. Paradise, Jared M. Evans, Jorge Torres-Mora, Marco Rizzo, Peter Kloen, Marianna Kruithof-de Julio, Andre J. van Wijnen, Sanjeev Kakar

**Affiliations:** Department of Orthopedic Surgery, Mayo Clinic, 200 First Street SW, Rochester, MN 55905 USA; Department of Anatomic Pathology, Mayo Clinic, Rochester, MN USA; Department of Biomedical Statistics and Informatics, Mayo Clinic Rochester, Rochester, MN USA; Department of Orthopedic Surgery, Academic Medical Center, Amsterdam, The Netherlands; Department of Urology, Leiden University Medical Center, Leiden, The Netherlands

Following the publication of the article in *BMC Medical Genomics* [1], we were made aware that the name of Marianna Kruithof – de Julio was incorrectly captured in the metadata.

Kruithof-de was incorrectly added to the first name, while it should have been captured as a part of the last name.

The first name of the author is: Marianna. The last name of the author is: Kruithof-de Julio. We apologize for the inconvenience this may have caused.

